# Persistence of Anti-SARS-CoV-2 Antibodies in Long Term Care Residents Over Seven Months After Two COVID-19 Outbreaks

**DOI:** 10.3389/fimmu.2021.775420

**Published:** 2022-01-03

**Authors:** Guadalein Tanunliong, Aaron Liu, Rohit Vijh, Tamara Pidduck, Jesse Kustra, Ana Citlali Márquez, Alexandra Choi, Meghan McLennan, Althea Hayden, Christy Kearney, Soren Gantt, Mel Krajden, Muhammad Morshed, Agatha N. Jassem, Inna Sekirov

**Affiliations:** ^1^ Department of Pathology and Laboratory Medicine, University of British Columbia, Vancouver, BC, Canada; ^2^ Department of Experimental Medicine, University of British Columbia, Vancouver, BC, Canada; ^3^ Office of the Chief Medical Health Officer, Vancouver Coastal Health, Vancouver, BC, Canada; ^4^ School of Population and Public Health, University of British Columbia, Vancouver, BC, Canada; ^5^ British Columbia Centre for Disease Control (BCCDC) Public Health Laboratory, Vancouver, BC, Canada; ^6^ Haro Park Centre, Vancouver, BC, Canada; ^7^ Centre de Recherche de Centre Hospitalier Universitaire (CHU) Sainte-Justine, Département de microbiologie, infectiologie et immunologie, Université de Montréal, Montréal, QC, Canada

**Keywords:** SARS-CoV-2, COVID-19, serologic testing, outbreak investigation, humoral immune response, long term care facilities, human coronavirus (HCoV)

## Abstract

**Background:**

As part of the public health outbreak investigations, serological surveys were carried out following two COVID-19 outbreaks in April 2020 and October 2020 in one long term care facility (LTCF) in British Columbia, Canada. This study describes the serostatus of the LTCF residents and monitors changes in their humoral response to SARS-CoV-2 and other human coronaviruses (HCoV) over seven months.

**Methods:**

A total of 132 serum samples were collected from all 106 consenting residents (aged 54-102) post-first outbreak (N=87) and post-second outbreak (N=45) in one LTCF; 26/106 participants provided their serum following both COVID-19 outbreaks, permitting longitudinal comparisons between surveys. Health-Canada approved commercial serologic tests and a pan-coronavirus multiplexed immunoassay were used to evaluate antibody levels against the spike protein, nucleocapsid, and receptor binding domain (RBD) of SARS-CoV-2, as well as the spike proteins of HCoV-229E, HCoV-HKU1, HCoV-NL63, and HCoV-OC43. Statistical analyses were performed to describe the humoral response to SARS-CoV-2 among residents longitudinally.

**Findings:**

Survey findings demonstrated that among the 26 individuals that participated in both surveys, all 10 individuals seropositive after the first outbreak continued to be seropositive following the second outbreak, with no reinfections identified among them. SARS-CoV-2 attack rate in the second outbreak was lower (28.6%) than in the first outbreak (40.2%), though not statistically significant (P>0.05). Gradual waning of anti-nucleocapsid antibodies to SARS-CoV-2 was observed on commercial (median Δ=-3.7, P=0.0098) and multiplexed immunoassay (median Δ=-169579, P=0.014) platforms; however, anti-spike and anti-receptor binding domain (RBD) antibodies did not exhibit a statistically significant decline over 7 months. Elevated antibody levels for beta-HCoVs OC43 (P<0.0001) and HKU1 (P=0.0027) were observed among individuals seropositive for SARS-CoV-2 compared to seronegative individuals.

**Conclusion:**

Our study utilized well-validated serological platforms to demonstrate that humoral responses to SARS-CoV-2 persisted for at least 7 months. Elevated OC43 and HKU1 antibodies among SARS-CoV-2 seropositive individuals may be attributed to cross reaction and/or boosting of humoral response.

## Introduction

Long term care facilities (LTCF) have been disproportionately affected by the coronavirus disease 2019 (COVID-19) pandemic. The high risk for respiratory virus transmission and outbreaks within the LTCF setting, in addition to advanced age and multiple co-morbidities, predisposes LTCF residents to a greater susceptibility to severe COVID-19 (1). In British Columbia, one LTCF experienced two COVID-19 outbreaks in April 2020 and October 2020. A lower prevalence following the second outbreak investigation in comparison to the first may be attributed to the refinement of public health infection control measures and the persistence of protective immune responses following the first outbreak (2). Additionally, pre-existing immunity to endemic coronaviruses may also be a contributing factor to the immune response development during SARS-CoV-2 infection.

Severe acute respiratory syndrome coronavirus 2 (SARS-CoV-2), the etiologic agent of COVID-19, emerged in December 2019 and poses an acute public health challenge worldwide; however, seasonal human coronaviruses (HCoV) are endemic and have long been recognized as the cause of ~10-30% of upper respiratory tract infections (3). The endemic HCoVs OC43 and HKU1 (beta-lineage coronavirus) along with NL63 and 229E (alpha-lineage coronavirus) exhibit sequence and structural homology to SARS-CoV-2 (4). Additionally, growing evidence demonstrates cross-reactivity between antibodies against endemic HCoV and SARS-CoV-2 (5). As such, there is interest in understanding the possibility that pre-existing immunity from endemic HCoVs contributes to protection against SARS-CoV-2 infection.

Published findings from our previous serological survey following the first LTCF outbreak demonstrated that serological testing uncovered seropositive cases that were missed during the outbreak investigation (2). Our main objective in this study was to describe the serostatus of residents and monitor the changes in their humoral response to SARS-CoV-2 and endemic HCoVs following the second outbreak. To investigate the persistence of antibody responses over seven months, we compared the sero-survey findings post-first and post-second COVID-19 outbreaks in one LTCF in British Columbia, Canada. Findings may provide better insight into pre-existing protection against SARS-CoV-2 infection, help guide public health infection control measures, and inform vaccination implementation guidelines.

## Materials and Methods

### Study Participants

Sero-surveys were conducted after COVID-19 outbreaks were declared over in the affected LTCF. The first serological survey was administered on May 4^th^ to 14^th^ 2020, and venous blood specimens were collected from residents in the LTCF (N = 87) (2). A second post-outbreak sero-survey was conducted on December 22^nd^, 2020, following a second outbreak in the fall of 2020, and venous blood specimens were collected from residents in the same LTCF (N = 45).

All LTCF residents (or their substitute decision makers) were informed of the planned investigations, and verbal consent for sample collection and testing was secured from all participants (or their substitute decision makers) who were willing to participate in the study. The investigations in this study were carried out as part of our public health-driven outbreak investigations in LTCF affected by COVID-19 outbreaks, and findings served to inform improvements to our outbreak control strategies. As such, these investigations were part of our practice and undertaken within our practice mandate, institutional ethics review was not required for specimen collection. Serological testing on clinical platforms was done as regular clinical testing. Serological testing on the Meso Scale Discovery (MSD) platform and subsequent analysis was done as part of the assay validation and approved by REB protocol H20-01089.

### Nucleic Acid Amplification Tests (NAAT)

Nasopharyngeal swab samples were collected from participants during the two outbreaks in April 2020 and October 2020, in accordance with established clinical and infection control practices in the facilities during the time of each outbreak (practices evolved over time) and all residents were tested by NAAT for active SARS-CoV-2 infection. NAAT testing was performed in accredited clinical laboratories using validated laboratory-developed assay (6) and/or Roche cobas^®^ SARS-CoV-2 commercially available test (7).

### Health Canada-Approved Commercial Serology Assays

Serological testing of participants from the first survey was performed using 5 different commercially available SARS-CoV-2 antibody assays in our previous study (2). Sera obtained from the second serological survey were tested in accordance with established clinical protocols using three single-antigen chemiluminescent assays. Specimens were first screened by ADVIA Centaur XP SARS-CoV-2 Total Antibody (Siemens T; Siemens, USA) to detect total antibodies to SARS-CoV-2 S1 RBD. All reactive samples received supplementary testing by ARCHITECT i2000 SARS-CoV-2 IgG (Abbott IgG; Abbott, USA), and VITROS 7600 Anti-SARS-CoV-2 Total Antibody (Ortho T; Ortho Clinical Diagnostics, USA) assays, detecting antibodies against SARS-CoV-2 Nucleocapsid and S1 Spike respectively. All commercial serology assays were carried out according to manufacturers’ protocols (8).

### Meso Scale Discovery Multiplex Immunoassay

As an additional supplementary test, we utilized a highly sensitive multiplex electrochemiluminescent immunoassay from MSD for the simultaneous detection of antibodies to SARS-CoV-2 and other circulating endemic HCoVs (V-PLEX Coronavirus Panel 2). Multi-spot plates spotted with purified and dried antigens were used for the detection of the following antibodies: spike, S1 RBD, and nucleocapsid of SARS-CoV-2, and spike proteins from circulating alpha-HCoV (229E and NL63) and beta-HCoV (HKU1 and OC43). Assays were performed according to manufacturer’s protocol for V-PLEX Coronavirus Panel 2 (9).

Briefly, multi-spot plates were initially incubated with MSD Blocker A for 30 minutes, then washed off. Reference standard, controls, and specimens (sera diluted 1:5000 in Diluent 100) were then added and incubated on the plates for 2 hours. Following incubation, plates were washed, incubated with MSD SULFO-TAG Anti-Human IgG detection antibody for an hour, then washed again. Finally, MSD Gold Read Buffer B was added to the wells and the assay plate was immediately measured on the MSD QuickPlex SQ120 instrument. All incubation steps were carried out in room temperature with shaking at 700rpm, and all wash steps were performed three times with MSD Wash Buffer, prior to addition of the subsequent reagents.

Raw data generated was processed using MSD Discovery Workbench software (Version 4.0), then imported into RStudio (Version 1.2.5033) to interpret signal cut-off values. Cut-off thresholds for reactivity were provided by the manufacturer and are as follows: SARS-CoV-2 spike values above 1960 AU/mL, nucleocapsid values above 5000 AU/mL, and S1 RBD values above 538 AU/mL. Samples above cut-off values for at least two of SARS-CoV-2 S1 RBD, nucleocapsid, and spike were considered serologically reactive using this MSD immunoassay.

### Statistical Analysis

Attack rates were calculated by taking the percentage of seropositive participants relative to the total number of susceptible participants for each outbreak. Two attack rates were calculated for outbreak 2 as above, based on two assumptions: A) Individuals seropositive in survey 1 were protected from reinfection during outbreak 2 (10/35), and B) Individuals seropositive in survey 1 were susceptible to reinfection (10/45).

Descriptive statistics of the study population were summarized on R (Version 3.6.2) and RStudio (Version 1.2.5033), and non-parametric statistical tests were carried out using the *ggpubr* (Version 0.6.0) and *stats* (Version 3.6.2) packages to assess attack rates, differences between groups, and changes in humoral responses across surveys. Processed data from the MSD immunoassay was visualized using *ggplot2* (Version 3.3.3) and *ggpubr* packages on RStudio.

## Results

### Study Population

A total of 132 serum samples were collected from the 106 LTCF residents included in this study. 87 of the serum samples were collected following the first outbreak, while 45 were collected following the second outbreak. Among the 106 residents, 26 of them provided their serum samples following both the first and second outbreaks, thus permitting the longitudinal assessment of SARS-CoV-2 antibodies in this subset of residents. The median age of all study participants included was 85 years, and the age and sex distribution of the LTCF residents included in this study is described in **Supplementary Table 1**.

### Seroprevalence and Attack Rates of SARS-CoV-2 in LTCF

A summary of the sero-survey results from both clinical and MSD assays are provided in **Table 1**, with the corresponding NAAT and serology status of each resident listed in **Supplementary Table 2**. Lower attack rates were observed during the second outbreak compared to the first using both clinical and MSD platforms. Specifically, when calculated under the assumption that the individuals who seroconverted in outbreak 1 remain susceptible to reinfection during the second outbreak (10/45), the attack rate during the second outbreak (22.2%) appeared significantly lower than during the first outbreak (40.2%) (P = 0.036). However, when calculated under the assumption that the individuals who seroconverted in outbreak 1 were protected from reinfection during the second outbreak (10/35), the attack rate during the second outbreak (28.6%) was lower, but not significantly lower than during the first outbreak (40.2%) (P > 0.05). Overall, while there appeared to be slightly greater odds of seroconversion (First assumption: Odds Ratio = 1.68, 95% CI: 0.67-4.42; Second assumption: Odds Ratio = 2.34 95% CI: 0.976-6.01) following the first outbreak compared to the second outbreak, this was not found to be statistically significant. As the first sero-survey was conducted at the beginning of the pandemic, these individuals were presumed to be negative for SARS-CoV-2 prior to the outbreaks. Additionally, comparisons between clinical and MSD interpretations demonstrated a 98.7% (76/77) agreement with negative samples and a 98.2% (55/56) agreement with positive samples (**Supplementary Figure 1**).

**Table 1:** Summary of sero-survey results.

Assumption	Serology Platform	OB	Positive (N)	Negative (N)	Total (N)	Attack Rate	P-Value
A	Commercial	**1**	35	52	87	40.2%	X^2^, P = 0.2274
		**2**	10	25	35	28.6%	Fisher, P = 0.3002
		**Total**	45	77	122		OR = 1.68 (0.674-4.42)
	MSD	**1**	35	52	87	40.2%	X^2^, P = 0.3643
		**2**	11	24	35	31.4%	Fisher, P = 0.4137
		**Total**	46	76	122		OR = 1.46 (0.598-3.76)
B	Commercial	**1**	35	52	87	40.2%	X^2^, P = 0.0386*
		**2**	10	35	45	22.2%	Fisher, P = 0.0523
		**Total**	45	77	132		OR = 2.34 (0.976-6.01)
	MSD	**1**	35	52	87	40.2%	X^2^, P = 0.0712
		**2**	11	34	45	32.4%	Fisher, P = 0.0845
		**Total**	46	76	132		OR = 2.06 (0.879-5.15)

Assumption A assumes that the 10 individuals seropositive from outbreak 1 were protected from reinfection and were excluded from the attack rate calculation. Assumption B assumes that reinfections are possible within the 10 seropositive from outbreak 1 and were susceptible to reinfection during outbreak 2. *p < 0.05. Bolded values initially indicated significance (p < 0.05).

**Figure 1:**
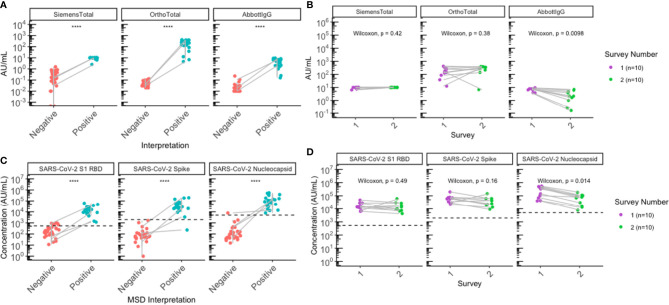
Sero-surveys identified three new seroconversions and demonstrated gradual waning of anti-nucleocapsid antibodies following the second outbreak. **(A, B)** Antibody levels for all residents with paired sera collected (N=26) were plotted by SARS-CoV-2 serostatus to assess SARS-CoV-2 antibodies. **(A)** Commercial serology data plotted according to clinical interpretation. **(B)** MSD data plotted according to MSD interpretation. **(C, D)** All participants (N=10) seropositive for SARS-CoV-2 following the first outbreak (purple) remained seropositive following the second outbreak (green) on both **(C)** commercial and **(D)** MSD platforms. Grey lines indicate paired samples collected from the same individual from both surveys, and lines on **(A, B)** traversing across negative (red) to positive (blue) indicate seroconversion, while vertical lines indicate that the individual’s paired serum samples both remained seronegative or seropositive across the two surveys. **(B, D)** Black dashed lines on represent positive signal cut-off for SARS-CoV-2 S1 RBD (538 AU/mL), spike (1960 AU/mL), and nucleocapsid (5000 AU/mL). Statistical analysis was performed using **(A, B)** Wilcoxon’s Rank Sum Test and **(C, D)** Wilcoxon’s Signed Rank Test. ****p<=0.0001.

A breakdown of the characteristics of the seropositive individuals is shown in **Table 2**. Following the first outbreak, 85.7% (30/35) had a previous positive NAAT test and 14.3% (5/35) of the seropositive cases had no previous NAAT test, as described previously (2). Serological testing was repeated following the second outbreak and 3 of the 26 paired residents were new seroconversions, all of whom were previously confirmed seronegative during the first survey, diagnosed with COVID-19 by NAAT during the second outbreak and identified as seropositive by both commercial serology (**Figure 1A**) and using MSD (**Figure 1B**). Notably, one additional seroconversion was identified by MSD (**Figure 1B**), where the individual was found reactive on MSD for anti-RBD and anti-nucleocapsid antibodies to SARS-CoV-2, but was non-reactive for either antigen on commercial platforms and had no positive NAAT history. No differences were observed in SARS-CoV-2 antibody levels between sexes (**Supplementary Figure 2**). Additionally, all 10 individuals seropositive during the first outbreak remained seropositive and had a negative NAAT test during the second outbreak, indicating no reinfections were identified during the second outbreak.

**Table 2:** Breakdown of seropositive participants based on clinical serology tests.

	N (%)
**Outbreak 1**	87
** Seropositive**	35 (100%)
Negative NAAT result	0 (0%)
Positive NAAT result	30 (85.7%)
No NAAT result	5 (14.3%)
**Outbreak 2**	45
** Seropositive**	20 (100%)
Seroconverted in outbreak 1	10 (50%)
Seroconverted in outbreak 2	3 (15%)
No baseline serology results from outbreak 1	7 (35%)

N, Number of participants tested; OB, Outbreak; N/A, Not Available.

### Duration of SARS-CoV-2 IgG

Individuals positive for SARS-CoV-2 during the first outbreak demonstrated statistically significant waning of anti-nucleocapsid IgG antibodies for SARS-CoV-2 between the first survey and the second survey in both the clinical Abbott IgG assay (median Δ = -3.7, P = 0.0098) (**Figure 1C**), as well as the MSD immunoassay (median Δ = -169579, P=0.014) (**Figure 1D**). Anti-spike and anti-RBD IgG antibodies remained relatively stable when assessed by clinical tests (**Figure 1C**), but a slight decrease was observed on the MSD assays (**Figure 1D**); however, none of these changes were statistically significant (**Figure 1C, D**; **Supplementary Table 3**). Notably, two seropositive individuals were found to have anti-nucleocapsid IgG below reactivity cut-off on MSD. One of the participants was non-reactive for anti-nucleocapsid on both commercial and MSD platforms post-first survey, the other individual was reactive for anti-nucleocapsid on commercial tests but non-reactive on MSD, just below the cut-off (4958 AU/mL) post-second survey.

### Elevation of HCoV-HKU1 and HCoV-OC43 Antibody Levels Among SARS-CoV-2 Positive Persons

**Figure 2:**
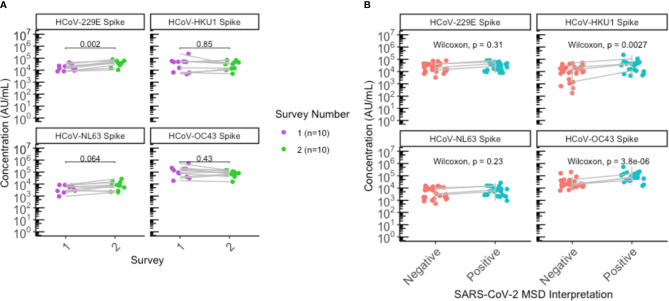
Significant elevation of HKU1 and OC43 antibodies in SARS-CoV-2 positive individuals. **(A)** HCoV antibody levels of residents that were seropositive during the first survey (N=10) were plotted according to the first (purple) and second (green) sero-surveys. **(B)** Antibody levels for all residents with paired sera collected (N=26) were plotted by SARS-CoV-2 negative (red) or positive (blue) status to assess antibody levels to endemic HCoV. Grey lines indicate paired samples collected from the same individual from both surveys, and lines on **(A, B)** traversing across negative (red) to positive (blue) indicate seroconversion, while vertical lines indicate that the individual’s paired serum samples both remained seronegative or seropositive across the two surveys. **(A, B)** Statistical analysis was performed using **(A)** Wilcoxon signed-rank test and **(B)** Wilcoxon rank-sum test.

Overall, antibody levels to all 4 endemic HCoV (229E, NL63, HKU1, OC43) spike proteins appeared relatively stable between the first and second sero-surveys (**Figure 2A**). Interestingly, anti-spike IgG for 229E was elevated (P = 0.002) following the second outbreak in comparison to the first outbreak, when looking at antibody levels for the 10 individuals that remained seropositive since the first outbreak (**Figure 2A**). While there appeared to be a slight difference in OC43 and HKU1 antibody levels between sexes (**Supplementary Figure 2**), the 10 individuals comprised of 9 females and 1 male; hence, no sex-stratified analysis can be performed.

Individuals seropositive for SARS-CoV-2 were also found to have elevated antibody levels for beta-HCoVs OC43 (P < 0.0001) and HKU1 (P = 0.0027), suggesting an immunologic boosting effect following SARS-CoV-2 infection and/or cross-reaction of anti-SARS-CoV-2 antibodies with beta-HCoV antibodies (**Figure 2B**; **Supplementary Figure 1**).

## Discussion

Our study evaluated the serostatus of LTCF residents and monitored changes in their humoral response and antibody titers over time. The lower attack rate and absence of reinfections observed during the second LTCF outbreak may be attributed to protective effects from herd immunity and/or enhanced public health infection control measures. Additionally, the COVID-19 related case fatality in the facility was 23.6% (13 of 55 residents) during the first outbreak and 6.7% (1 of 15 residents) during the second outbreak, and while our study only included residents that consented to participating in this sero-survey, the lower case fatality during the second outbreak also supports that improved infection control measures and persistent antibody response from the first outbreak (along with improved management of COVID-19 disease by the medical community over the course of the pandemic) may have contributed to the lower attack rate and absence of reinfections during the second outbreak. Moreover, unlike the first outbreak, no additional cases of SARS-CoV-2 infection were uncovered by serological testing post second outbreak. While the MSD assay indicated one additional seroconversion, this individual is less likely to be a true positive because, while they were reactive for anti-RBD and anti-nucleocapsid on MSD, they were non-reactive on commercial assays and have negative NAAT results during the outbreaks. Additionally, reactivity with SARS-CoV-2 RBD and nucleocapsid by the MSD assay could have been due to cross-reactivity from high levels of endemic HCoV antibodies found in this individual’s serum.

Due to the recent emergence of SARS-CoV-2, there are limited longitudinal immunologic studies investigating the stability of SARS-CoV-2 and HCoV antibody levels across various population demographics over a longer duration (10–12). Our findings suggest that a SARS-CoV-2 infection in the elderly was able to elicit an immune response lasting at least 7 months. Here, we found persistent antibody titers for SARS-CoV-2 spike and RBD between the first and the second sero-surveys for the 10 individuals infected during the first outbreak, but a statistically significant waning of SARS-CoV-2 nucleocapsid antibodies among these individuals was observed by 2 serological assays. Our results are consistent with multiple published literature describing similar trends, where nucleocapsid antibodies demonstrated faster waning and a shorter half-life, frequently falling to below detectable levels within 4-7 months (12–15). Notably, the individuals from our study all remained seropositive after 7 months and had detectable levels of anti-nucleocapsid antibodies present, despite the significant waning of anti-nucleocapsid IgG levels observed.

There have also been studies that reported declining anti-spike and anti-RBD IgG titers over time. Similar to our study, Dan et al. and Cohen et al. also evaluated SARS-CoV-2 immune responses longitudinally in COVID-19 patients over 6 to 8 months (16, 17). While our results all agree that anti-Spike and anti-RBD were longer lasting than anti-nucleocapsid antibodies in COVID-19 patients, both Dan et al. and Cohen et al. utilized half-life modeling to predict declining antibody kinetics in the general adult population. As described in our **Supplementary Table 3**, our study also demonstrated a declining trend in antibody titers among these 10 individuals for RBD (median Δ=-2906.7, P=0.49) and Spike (median Δ=-24567.3, P = 0.16) using the MSD assay, although unlike Nucleocapsid (median Δ=-169579, P=0.014), we did not find this to be statistically significant.

The differences in antibody durability and protection against reinfection may be attributed to various factors including cohort characteristics and COVID-19 severity (14, 18). Dan et al. found that 90% (36/40) of subjects remained seropositive for Spike IgG while 88% (35/40) remained positive for RBD IgG after 6 to 8 months (16). In contrast, we saw all 10 individuals remain seropositive over the 7 months. As our study was conducted as part of the public health outbreak investigations, we were limited to 10 individuals that were seropositive during the first outbreak and consented to participating in both first and second surveys for our longitudinal comparisons in the same individual. It is possible that observing a larger population over time would allow us to see the same proportion of seropositive individuals that Dan et al. saw in the specified timeframe (16). Interestingly, those with older age and severe COVID-19 were found to exhibit stronger immune responses, higher IgG titers, and seroconvert to negative more slowly than those with mild or asymptomatic disease (14, 17, 19). With this in mind, the 10 individuals in our study comprised of older aged adults that are likely to have more severe disease or worse clinical progression, which may contribute to slower waning in this population in contrast to other studies that evaluated immune durability in healthy adults that primarily have mild or asymptomatic disease (16, 17). As such, the disparity in findings reported across multiple studies investigating the persistence of SARS-CoV-2 antibodies longitudinally is likely attributed to various cohort-specific factors.

Alternatively, the timing of an individual’s seroconversion may also contribute to the disparity in findings across published studies on antibody durability. Specifically, the first survey was conducted in May 2020, a month following the facility outbreak in April 2020. Previous studies have shown that it can take 4-5 weeks to reach peak antibody responses following a primary infection (20), and this may even take months in those with severe disease (21). There are studies that describe older age as a predictor of poor seroconversion (22), perhaps due to potential comorbidities or immunosenescence. As such, it is possible that some of the 10 individuals that were seropositive during the first survey had antibody responses that remained on the rising phase and have yet to reach peak IgG levels by the time of the first survey. In this case, if the true decay from peak IgG levels was missed, this may potentially explain the absence of a significant decline in SARS-CoV-2 RBD and Spike antibody levels among our elderly population. This highlights the importance of better understanding how various biological factors may contribute to variation in seroconversion rates and antibody dynamics within the population.

Serological assays do not directly quantify neutralizing antibodies; however, they have been shown to correlate with levels of neutralizing antibodies and may be indicative of protection against reinfection (23). While emerging data has shown that previous infection is not a guarantee for immunity from reinfection (24), our data demonstrated that all residents known to be seropositive post-first outbreak who were also included in the second survey remained seropositive for SARS-CoV-2 antibodies but negative on NAAT, thus providing evidence that a pre-existing infection-induced serological response to SARS-CoV-2 may contribute protection against reinfection in the elderly for at least 7 months. Notably, 2/10 residents seropositive during the first outbreak appeared to show higher anti-Spike and anti-RBD antibody titers during the second outbreak, but not for nucleocapsid (**Figure 1**). It is possible that this boosting could be attributed to a potential recall response, where re-exposure of these 2 individuals to SARS-CoV-2 during the second outbreak may have not been sufficient to establish a clinically detectable reinfection (NAAT negative), but was enough to elicit a serological memory response that was detectable through sensitive serologic testing. This further supports that previous immunity may offer some protection from reinfection, although more studies need to be conducted to evaluate the degree of protection across individuals, given the heterogeneity of immune responses to SARS-CoV-2 in the population. Taken together, our findings are also consistent with recent evidence showing an 84% reduction in risk of reinfection when observed 7 months following primary infection (25). It has been conclusively shown that vaccine-induced humoral responses are stronger than those from natural infection, and vaccination is superior to natural immunity in protecting from infection (26). However, better understanding of the persistence versus waning dynamic of infection-induced responses may help in allocation of primary and booster vaccine doses in settings where these resources are scarce.

By utilizing a multiplexed immunoassay, we were able to simultaneously detect endemic HCoV antibodies in addition to SARS-CoV-2 antibodies. We found anti-spike IgG to endemic HCoV appear relatively stable overall between the first and second surveys, with a slight elevation in levels of 229E antibodies post-second survey among those seropositive for SARS-CoV-2 from the first survey. While our results found this difference to be statistically significant, there has been limited literature on the relationship between humoral responses to SARS-CoV-2 and alpha-HCoVs and this difference could likely be attributed to our small sample size and intra-assay variability. Additionally, there has been no known HCoV outbreak in British Columbia in the past two years, suggesting that the elevated levels of 229E spike antibodies are unlikely due to recent exposures. Nonetheless, the observed difference in 229E antibody levels can be explored further using larger sample sizes and in the general adult population.

Consistent with multiple published findings (4, 19, 27, 28), we also observed significantly elevated endemic antibody levels for beta-HCoVs OC43 and HKU1 following SARS-CoV-2 infection and seroconversion, unlike alpha-HCoVs. This finding is not unexpected due to the higher sequence and structural homology of beta-HCoVs with SARS-CoV-2 (29). It is unknown whether cross-reactivity or immunologic boosting related to OC43 and HKU1 antibody responses may contribute to protection against COVID-19. Previous studies suggest that endemic HCoV antibodies have poor neutralizing activity against SARS-CoV-2 (4, 30), while other studies describe that higher levels of HCoV-OC43 and HCoV-HKU1 antibodies are associated with less severe course of disease (31, 32). Nonetheless, the clinical significance of pre-existing immunity from endemic HCoV exposure against SARS-CoV-2 infection and COVID-19 severity remains to be determined.

In summary, our study used well-validated assays to provide evidence that humoral responses to SARS-CoV-2 elicited by natural infection persist over at least seven months in the elderly and may confer protection from reinfection. Taken together, our results also suggest the potential effectiveness of the quality improvement efforts in infection control after the first outbreak (2). Limitations of our study include a small sample size of residents from a single LTCF; therefore, it may not be representative of other elderly populations with varying demographic characteristics (i.e., age, sex, geographic region, etc.). Additionally, a natural survival bias is intrinsic to this type of survey, where serological testing is conducted following the outbreaks, as such, those captured in either outbreak would have had to survive to be included in our dataset. Including those that did not participate in our serosurveys, the case fatality during the first and second outbreak was 23.6% (13/55) and 6.7% (1/15) respectively. The lower case fatality during the second outbreak is likely multifactorial in nature. While this does not directly address survival bias, similar to the lower attack rate during the second survey, this decreasing trend could also be attributed to improved infection control measures in the facility following the first outbreak, although additional contributions from improved management of COVID-19 by the time of second outbreak (due to improved knowledge in the medical field) is also likely to contribute. Furthermore, our study only assessed IgG responses longitudinally, which are expected to persist for a longer duration. Recent findings have also demonstrated that SARS-CoV-2 IgA antibodies can persist for over 6 months following infection (16, 32–34), suggesting that quantifying IgA antibodies may potentially be beneficial to sero-surveillance studies and add to its overall sensitivity in identifying past infections. Future studies evaluating other SARS-CoV-2 and HCoV antibody isotypes longitudinally may provide valuable insight in understanding the humoral response to SARS-CoV-2 infections.

The strength of our study includes using multiple serological platforms that are authorized for diagnostic use, as well as MSD’s pan-coronavirus multiplex immunoassay that allows for highly sensitive simultaneous detection of IgG against SARS-CoV-2 and HCoV antigens, thus we have the advantage of reporting consistent data reproducible across different platforms that are known to be highly reliable. Additionally, outbreaks in LTCF remain a major concern in the proper management of the COVID-19 pandemic due to higher burden of disease and risk of severe complications among the elderly. Therefore, while plenty of studies investigate the antibody responses longitudinally in the healthy adult population, our study contributes substantially to the field and fills the gap in knowledge of understanding how antibody response change and persist over time in older, high-risk adults.

The findings of our study provide insight into humoral responses to SARS-CoV-2 among LTCF residents and the potential for reinfection. However, additional studies are needed to more extensively characterize the durability of SARS-CoV-2 immune responses following natural infection and vaccination, and their role in protection against SARS-CoV-2 infection and COVID-19 severity among high-risk groups. In particular, large-scale longitudinal studies from multiple LTCF would provide valuable data on the durability of immune responses among institutionalized older adults.

## Data Availability Statement

The raw data supporting the conclusions of this article will be made available by the authors, without undue reservation.

## Ethics Statement

The investigations in this study were carried out as part of our public health-driven outbreak investigations and and undertaken within our practice mandate. Institutional ethics review was not required for specimen collection. Written informed consent for participation was not required for this study in accordance with the national legislation and the institutional requirements. Serological testing on clinical platforms was performed as regular clinical testing. Serological testing on the Meso Scale Discovery (MSD) platform and subsequent analysis was done as part of the assay validation and approved by REB protocol H20-01089.

## Author Contributions

GT, AL, RV, AC, AH, MMo, AJ, and IS were involved in the study conceptualization and methodology. Data curation was conducted by GT, AL, RV, TP, and JK, and data analysis was performed by GT, AL, and RV. CM assisted in sample management. CK carried out the recruitment of study participants. MMc was involved in experimental organization and sample collection. MK and SG were involved in the supervision of the study. The manuscript was originally drafted by GT and edited by IS, AJ, SG, TP, AC, and MMc. All authors contributed to the article and approved the submitted version.

## Funding

This work was funded by Genome British Columbia (Grant number COV050). The study sponsor had no role in data collection, analysis, and interpretation.

## Conflict of Interest

SG reports having received research support and/or consulting fees from Moderna, Merck, GSK, VBI Vaccines, and Meridian Biosciences. No products of these companies were used in the study.

The remaining authors declare that the research was conducted in the absence of any commercial or financial relationships that could be construed as a potential conflict of interest.

## Publisher’s Note

All claims expressed in this article are solely those of the authors and do not necessarily represent those of their affiliated organizations, or those of the publisher, the editors and the reviewers. Any product that may be evaluated in this article, or claim that may be made by its manufacturer, is not guaranteed or endorsed by the publisher.
