# Supplementary material for: Persistence of Anti-SARS-CoV-2 Antibodies in Long Term Care Residents Over Seven Months After Two COVID-19 Outbreaks
Source: Front Immunol. 2022 Jan 3;12:775420. doi: 10.3389/fimmu.2021.775420 (PMC8763385; doi:10.3389/fimmu.2021.775420)
Supplement: Supplementary file 1 [file Table_1.docx]

Supplementary Material

# Supplementary Material

**Supplementary Table 1. Age and Sex Distribution of Study Participants. N = total number of individuals. SD = standard deviation**

|  | **First sero-survey (N = 87)** | | **Second sero-survey (N = 45)** | |
| --- | --- | --- | --- | --- |
|  | **N (%)** | **Median Age (SD)** | **N (%)** | **Median Age (SD)** |
| **Sex** |  |  |  |  |
| Female | 58 (66.7) | 87.0(9.1) | 28 (62.2) | 89.0(7.5) |
| Male | 28 (32.2) | 78.0(8.7) | 14 (31.1) | 80.5(6.9) |
| Unknown | 1 (1.1) | 91 | 3 (6.7) | 86.0(4.7) |
| **Total** | 87 (100) | 84(9.7) | 45 (100) | 86(7.8) |
