# Supplementary material for: Persistence of Anti-SARS-CoV-2 Antibodies in Long Term Care Residents Over Seven Months After Two COVID-19 Outbreaks
Source: Front Immunol. 2022 Jan 3;12:775420. doi: 10.3389/fimmu.2021.775420 (PMC8763385; doi:10.3389/fimmu.2021.775420)
Supplement: Supplementary file 2 [file Table_2.docx]

Supplementary Material

# Supplementary Material

**Supplementary Table 2. NAAT and Serology Status of All 106 Residents included in both LTCF outbreaks.**

|  |  | **Outbreak 1 (N = 87)** | | | **Outbreak 2 (N = 45)** | | |  |
| --- | --- | --- | --- | --- | --- | --- | --- | --- |
| **Patient ID** | **Age** | **Clinical Serology** | **MSD Serology** | **NAAT** | **Clinical Serology** | **MSD Serology** | **NAAT** | **Alive at the end of study** |
| 1 | 93 | Positive | Positive | Positive | Positive | Positive | Negative | Yes |
| 2 | 69 | Positive | Positive | Positive | - | - | - | - |
| 3 | 90 | Positive | Positive | Positive | Positive | Positive | Negative | Yes |
| 4 | 82 | Positive | Positive | Positive | - | - | - | - |
| 5 | 90 | Positive | Positive | Positive | - | - | - | - |
| 6 | 85 | Negative | Negative | Negative | Negative | Negative | Negative | Yes |
| 7 | 88 | Positive | Positive | Positive | - | - | - | - |
| 8 | 85 | Positive | Positive | - | - | - | - | - |
| 9 | 97 | Negative | Negative | Negative | Negative | Negative | Negative | Yes |
| 10 | 77 | Negative | Negative | Negative | - | - | - | - |
| 11 | 77 | Negative | Negative | Negative | - | - | - | - |
| 12 | 92 | Positive | Positive | Positive | - | - | - | - |
| 13 | 72 | Positive | Positive | Positive | - | - | - | - |
| 14 | 86 | Positive | Positive | Positive | - | - | - | - |
| 15 | 84 | Negative | Negative | Negative | - | - | - | - |
| 16 | 96 | Negative | Negative | Negative | - | - | - | - |
| 17 | 91 | Positive | Positive | Positive | - | - | - | - |
| 18 | 81 | Negative | Negative | Negative | - | - | - | - |
| 19 | 89 | Positive | Positive | Positive | Positive | Positive | Negative | Yes |
| 20 | 67 | Negative | Negative | Negative | Negative | Negative | Negative | Yes |
| 21 | 87 | Negative | Negative | Negative | - | - | - | - |
| 22 | 90 | Positive | Positive | Positive | Positive | Positive | Negative | Yes |
| 23 | 87 | Negative | Negative | Negative | Negative | Positive | Negative | Yes |
| 24 | 80 | Negative | Negative | Negative | Positive | Positive | Positive | Yes |
| 25 | 83 | Positive | Positive | Positive | Positive | Positive | Negative | Yes |
| 26 | 69 | Negative | Negative | Negative | - | - | - | - |
| 27 | 81 | Positive | Positive | Positive | - | - | - | - |
| 28 | 73 | Negative | Negative | Negative | - | - | - | - |
| 29 | 81 | Negative | Negative | Negative | Negative | Negative | Negative | Yes |
| 30 | 97 | Negative | Negative | Negative | - | - | - | - |
| 31 | 90 | Positive | Positive | Positive | Positive | Positive | Negative | Yes |
| 32 | 98 | Negative | Negative | Negative | - | - | - | - |
| 33 | 93 | Negative | Negative | Negative | - | - | - | - |
| 34 | 91 | Negative | Negative | Negative | - | - | - | - |
| 35 | 74 | Negative | Negative | Negative | - | - | - | - |
| 36 | 86 | Negative | Negative | Negative | Positive | Positive | Positive | Yes |
| 37 | 69 | Positive | Positive | - | Positive | Positive | Negative | Yes |
| 38 | 65 | Negative | Negative | Negative | - | - | - | - |
| 39 | 73 | Negative | Negative | Negative | - | - | - | - |
| 40 | 91 | Negative | Negative | Negative | - | - | - | - |
| 41 | 78 | Negative | Negative | Negative | Positive | Positive | Positive | Yes |
| 42 | 77 | Negative | Negative | Negative | - | - | - | - |
| 43 | 94 | Positive | Positive | Positive | - | - | - | - |
| 44 | 83 | Negative | Negative | Negative | - | - | - | - |
| 45 | 102 | Positive | Positive | Positive | - | - | - | - |
| 46 | 73 | Negative | Negative | Negative | - | - | - | - |
| 47 | 86 | Negative | Negative | Negative | Negative | Negative | Negative | Yes |
| 48 | 71 | Negative | Negative | Negative | - | - | - | - |
| 49 | 74 | Negative | Negative | Negative | - | - | - | - |
| 50 | 88 | Negative | Negative | Negative | Negative | Negative | Negative | Yes |
| 51 | 73 | Positive | Positive | Positive | - | - | - | - |
| 52 | 81 | Positive | Positive | Positive | Positive | Positive | Negative | Yes |
| 53 | 82 | Positive | Positive | Positive | - | - | - | - |
| 54 | 81 | Positive | Positive | Positive | - | - | - | - |
| 55 | 79 | Negative | Negative | Negative | Negative | Negative | Negative | Yes |
| 56 | 69 | Positive | Positive | - | - | - | - | - |
| 57 | 92 | Negative | Negative | Negative | - | - | - | - |
| 58 | 74 | Negative | Negative | Negative | - | - | - | - |
| 59 | 90 | Negative | Negative | Negative | Negative | Negative | Negative | Yes |
| 60 | 96 | Positive | Positive | Positive | - | - | - | - |
| 61 | 85 | Positive | Positive | Positive | - | - | - | - |
| 62 | 82 | Negative | Negative | Negative | - | - | - | - |
| 63 | 69 | Positive | Positive | - | - | - | - | - |
| 64 | 66 | Negative | Negative | Negative | - | - | - | - |
| 65 | 76 | Negative | Negative | Negative | Negative | Negative | Negative | Yes |
| 66 | 78 | Positive | Positive | Positive | Positive | Positive | Negative | Yes |
| 67 | 54 | Positive | Positive | - | - | - | - | - |
| 68 | 87 | Positive | Positive | Positive | - | - | - | - |
| 69 | 97 | Negative | Negative | Negative | - | - | - | - |
| 70 | 72 | Negative | Negative | Negative | - | - | - | - |
| 71 | 93 | Negative | Negative | Negative | - | - | - | - |
| 72 | 97 | Positive | Positive | Positive | - | - | - | - |
| 73 | 87 | Negative | Negative | Negative | - | - | - | - |
| 74 | 78 | Positive | Positive | Positive | - | - | - | - |
| 75 | 87 | Negative | Negative | Negative | Negative | Negative | Negative | Yes |
| 76 | 84 | Negative | Negative | Negative | - | - | - | - |
| 77 | 77 | Negative | Negative | Negative | - | - | - | - |
| 78 | 89 | Negative | Negative | Negative | Negative | Negative | Negative | Yes |
| 79 | 67 | Negative | Negative | Negative | - | - | - | - |
| 80 | 92 | Positive | Positive | Positive | Positive | Positive | Negative | Yes |
| 81 | 95 | Negative | Negative | Negative | Negative | Negative | Negative | Yes |
| 82 | 78 | Negative | Negative | Negative | - | - | - | - |
| 83 | 92 | Negative | Negative | Negative | - | - | - | - |
| 84 | 102 | Negative | Negative | Negative | - | - | - | - |
| 85 | 83 | Negative | Negative | Negative | - | - | - | - |
| 86 | 93 | Positive | Positive | Positive | - | - | - | - |
| 87 | 76 | Positive | Positive | Positive | - | - | - | - |
| 88 | 72 | - | - | - | Positive | Positive | Negative | Yes |
| 89 | 94 | - | - | - | Positive | Positive | Negative | Yes |
| 90 | 98 | - | - | - | Negative | Negative | Negative | Yes |
| 91 | 74 | - | - | - | Positive | Positive | Positive | Yes |
| 92 | 73 | - | - | - | Negative | Negative | Negative | Yes |
| 93 | 94 | - | - | - | Negative | Negative | Negative | Yes |
| 94 | 88 | - | - | - | Negative | Negative | Negative | Yes |
| 95 | 97 | - | - | - | Negative | Negative | Negative | Yes |
| 96 | 80 | - | - | - | Positive | Positive | Positive | Yes |
| 97 | 89 | - | - | - | Negative | Negative | Negative | Yes |
| 98 | 78 | - | - | - | Positive | Positive | Negative | Yes |
| 99 | 82 | - | - | - | Negative | Negative | Negative | Yes |
| 100 | 89 | - | - | - | Negative | Negative | Negative | Yes |
| 101 | 79 | - | - | - | Negative | Negative | Negative | Yes |
| 102 | 72 | - | - | - | Negative | Negative | Negative | Yes |
| 103 | 90 | - | - | - | Positive | Positive | Positive | Yes |
| 104 | 83 | - | - | - | Negative | Negative | Negative | Yes |
| 105 | 84 | - | - | - | Positive | Positive | Positive | Yes |
| 106 | 85 | - | - | - | Negative | Negative | - | Yes |
