# Supplementary material for: Persistence of Anti-SARS-CoV-2 Antibodies in Long Term Care Residents Over Seven Months After Two COVID-19 Outbreaks
Source: Front Immunol. 2022 Jan 3;12:775420. doi: 10.3389/fimmu.2021.775420 (PMC8763385; doi:10.3389/fimmu.2021.775420)
Supplement: Supplementary file 3 [file Table_3.docx]

Supplementary Material

# Supplementary Material

**Supplementary Table 3. Change in SARS-CoV-2 antibody levels following the first and second outbreaks for individuals who seroconverted in outbreak 1 and remained seropositive into outbreak 2. N = 10.**

|  | **Median Delta** | **P-value** |
| --- | --- | --- |
| **Commercial Serology** |  |  |
| Siemens (S1 RBD) | 0 | 0.42 |
| Ortho (Spike) | 93.9 | 0.38 |
| Abbott (Nucleocapsid) | -3.7 | 0.0098* |
| **MSD** |  |  |
| S1 RBD | -2906.7 | 0.49 |
| Spike | -24567.3 | 0.16 |
| Nucleocapsid | -169579.04 | 0.014* |
