# Supplementary material for: Persistence of Anti-SARS-CoV-2 Antibodies in Long Term Care Residents Over Seven Months After Two COVID-19 Outbreaks
Source: Front Immunol. 2022 Jan 3;12:775420. doi: 10.3389/fimmu.2021.775420 (PMC8763385; doi:10.3389/fimmu.2021.775420)
Supplement: Supplementary file 4 [file Image_1.pdf]

Supplementary Material

1 Supplementary Material

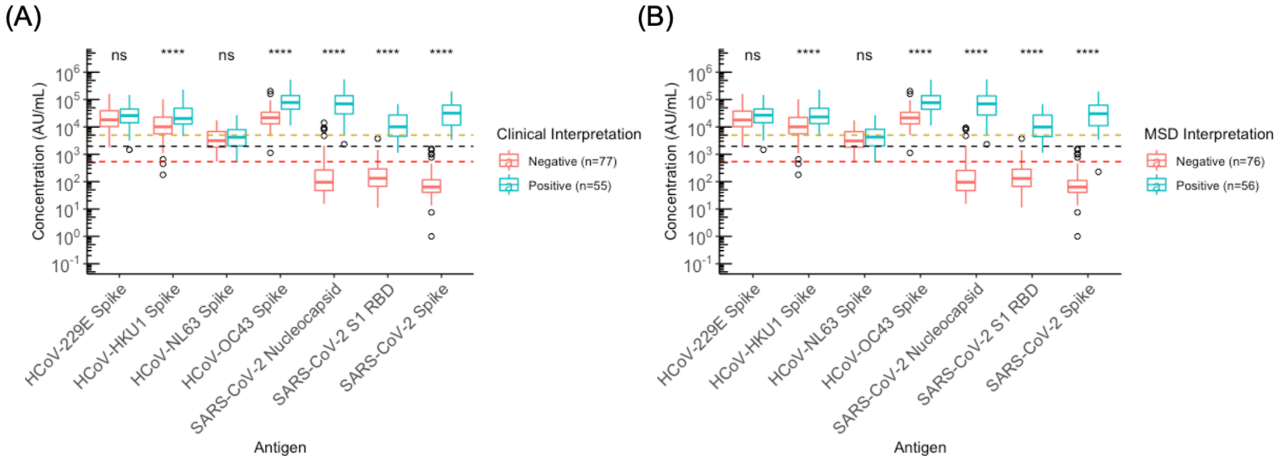

Supplementary Figure 1. Near-perfect agreement observed between clinical and MSD interpretations for negative and positive samples. MSD results plotted according to **(A)** clinical and **(B)** MSD interpretations demonstrate near perfect agreement between the platforms. Dashed lines represent positive signal cut-off for SARS-CoV-2 S1 RBD (red, 538 AU/mL), spike (black, 1938 AU/mL), and nucleocapsid (yellow, 5000 AU/mL). Notches on boxplots represent 95% confidence intervals. Black dots indicate outliers. Statistical analysis was performed using Wilcoxon rank-sum test. ns: $p>0.05$. ****: $p<=0.0001$.
