# Supplementary material for: Persistence of Anti-SARS-CoV-2 Antibodies in Long Term Care Residents Over Seven Months After Two COVID-19 Outbreaks
Source: Front Immunol. 2022 Jan 3;12:775420. doi: 10.3389/fimmu.2021.775420 (PMC8763385; doi:10.3389/fimmu.2021.775420)
Supplement: Supplementary file 5 [file Image_2.pdf]

Supplementary Material

1 Supplementary Material

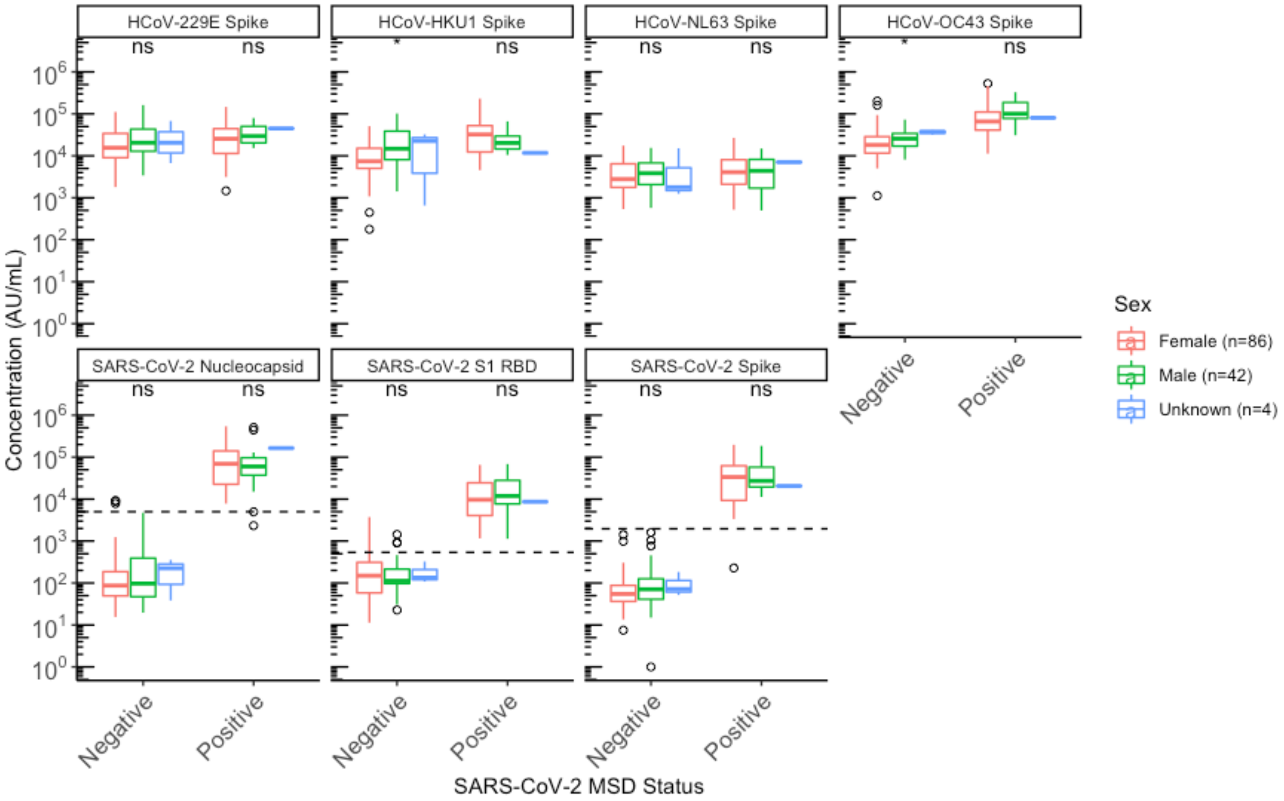

Supplementary Figure 2. HCoV and SARS-CoV-2 antibody levels by sex. MSD results for all serum samples (N = 132) plotted according to sex and SARS-CoV-2 MSD interpretations. Dashed lines represent positive signal cut-off for SARS-CoV-2 S1 RBD (538 AU/mL), spike (1938 AU/mL), and nucleocapsid (5000 AU/mL). Notches on boxplots represent 95% confidence intervals. Black dots indicate outliers. Statistical analysis was performed using Wilcoxon rank-sum test. ns: $p > 0.05$. *: $p < 0.05$.
